# Post Kala-Azar Dermal Leishmaniasis following Treatment with 20 mg/kg Liposomal Amphotericin B (Ambisome) for Primary Visceral Leishmaniasis in Bihar, India

**DOI:** 10.1371/journal.pntd.0002611

**Published:** 2014-01-02

**Authors:** Sakib Burza, Prabhat Kumar Sinha, Raman Mahajan, Marta González Sanz, María Angeles Lima, Gaurab Mitra, Neena Verma, Pradeep Das

**Affiliations:** 1 Médecins Sans Frontières, New Delhi, India; 2 Rajendra Memorial Research Institute of Medical Sciences, Patna, Bihar, India; 3 Médecins Sans Frontières, Barcelona, Spain; Emory University, United States of America

## Abstract

**Background:**

The skin disorder Post Kala-Azar Dermal Leishmaniasis (PKDL) occurs in up to 10% of patients treated for visceral leishmaniasis (VL) in India. The pathogenesis of PKDL is not yet fully understood. Cases have been reported in India following therapy with most available treatments, but rarely in those treated with liposomal amphotericin B (Ambisome). Between July 2007 and August 2012 with the support of the Rajendra Memorial Research Institute (RMRI), Médecins Sans Frontières (MSF) supported a VL treatment programme in Bihar, India—an area highly endemic for *Leishmania donovani*—in which 8749 patients received 20 mg/kg intravenous Ambisome as first-line treatment. This study describes the characteristics of patients who returned to the MSF supported treatment programme with PKDL.

**Methods and Principal Findings:**

Over a 5-year period, Ambisome was administered to 8749 patients with laboratory-confirmed VL (clinical signs, rK39 positive, with/without parasite confirmation) in four intravenous doses of 5 mg/kg to a total of 20 mg/kg, with a high initial-cure rate (99.3%) and low default rate (0.3%). All patients received health education highlighting the possibility and symptoms of developing PKDL, and advice to return to the MSF programme if these symptoms developed. This is an observational retrospective cohort study of the programme outcomes. Of the 8311 patients completing treatment for their first episode of VL, 24 (0.3%) returned passively to the programme complaining of symptoms subsequently confirmed as PKDL, diagnosed from clinical history, appearance consistent with PKDL, and slit-skin smear examination. Of the 24 patients, 89% had macular lesions, with a median time (interquartile range) to development of 1.2 (0.8–2.2) years following treatment. Comparison of the demographic and clinical characteristics of the VL patients treated with Ambisome who later developed PKDL, with those of the remaining cohort did not identify any significant risk factors for PKDL. However, the time to developing PKDL was significantly shorter with Ambisome than in a subset of patients presenting to the programme with PKDL following previous sodium stibogluconate treatment for VL.

**Conclusions:**

In this large cohort of patients with VL in Bihar who were treated with 20 mg/kg Ambisome, PKDL following treatment appears to be infrequent with no predictive risk factors. The shorter median time to developing symptoms of PKDL compared with that after conventional VL treatments should be taken into account when counseling patients treated with regimens including Ambisome.

## Author Summary

Visceral leishmaniasis (VL), also known as Kala-azar, is caused by the parasite *L.donovani*. Half of cases worldwide occur in India, with up to 90% of these in Bihar state. Post Kala-azar dermal leishmaniasis (PKDL) is a difficult to treat skin condition that develops in up to 10% of VL cases following treatment in the Indian subcontinent. Although often mild, PKDL can be severe and disfiguring. Patients are otherwise healthy. PKDL is considered a reservoir of *L.donovani* and requires treating to support disease elimination. Between 2007–2012, 8311 patients diagnosed with a first episode of VL completed treatment with 20 mg/kg intravenous liposomal amphotericin B (Ambisome) in a Médecins Sans Frontières (MSF) programme supported by the Rajendra Memorial Research Institute (RMRI) in Bihar. Ambisome is a safe and effective treatment that is recommended by the WHO for first-line use in the Indian subcontinent; although not yet included in the Indian guidelines. PKDL has been described following all VL treatments, but so far in only two patients treated with Ambisome. Here, we describe 24 patients treated with Ambisome who returned to the MSF treatment programme with confirmed PKDL. We found no risk factors for PKDL following treatment; however, the average time to development of PKDL was much shorter than that seen following older treatments.

## Introduction

The skin condition post Kala-azar dermal leishmaniasis (PKDL) usually develops following treatment for visceral leishmaniasis (VL), which is caused by the protozoa *Leishmania donovani*. Although cases of PKDL have been described in patients not previously diagnosed with or treated for VL, up to 10% of patients with VL in the Indian subcontinent go onto develop PKDL following treatment for symptomatic VL [1]. This proportion has been shown to be as high as 60% in Sudan [2] where, although the same species is responsible, the presentation of PKDL and its disease characteristics are different to that seen in the Indian subcontinent.

Little is known about the cause, risk factors, and pathogenesis of PKDL although host factors [3], in vivo hybridization of parasites [4], and the possibility that PKDL is a drug-related phenomenon [5] have all been considered. In the Indian context particularly, the low incidence of PKDL makes prospective studies challenging.

Traditionally, PKDL has been thought of as an important reservoir of *L. donovani* during intra-epidemic periods of VL, and may have been responsible for an epidemic of VL in an area of West Bengal [6]. Clarification of the potential transmissibility of different morphologies of PKDL remains an area of research priority [7].

Between July 2007 and August 2012, Médecins Sans Frontières (MSF), in coordination with the Rajendra Memorial Research Institute of Medical Sciences (RMRIMS; Patna, Bihar), the Bihar State Health Society, and the National Vector Borne Disease Control Programme of India treated 8311 patients diagnosed with a first episode of VL with 20 mg/kg intravenous liposomal amphotericin B (Ambisome; Gilead Pharmaceuticals, Foster City, CA, USA) as first-line treatment.

Ambisome is a brand name for Liposomal Amphotericin B. There are a number of preparations of Liposomal Amphotericin B available on the market; however due to the lack of standard and widely applicable regulations or guidance for liposomal technology, it is important that this specific preparation be named. At time of publication, none of the rival preparations have undergone peer reviewed non-inferiority studies against Ambisome nor received stringent regulatory approval for use in VL. It is for this reason that MSF and the WHO currently only use Ambisome rather than other preparations. However it is urgent that clear regulatory guidelines for endemic countries be established by a normative setting organisation like the WHO and other existing formulations be formally evaluated [8].

This is the largest cohort of VL patients treated with Ambisome worldwide. Here, we describe the characteristics of patients who re-attended the programme with confirmed PKDL. As far as the authors are aware, to date PKDL has been described following all VL treatments, but so far in only 2 patients treated with Ambisome in the Indian context [9].

## Methods

In coordination with the local partners detailed above, MSF developed an integrated programme of VL-case identification and treatment within existing government structures in Vaishali district, Bihar. Patients were diagnosed and treated either as outpatients at primary healthcare centers or as inpatients at the district hospital. Patients for whom the diagnosis was questionable were referred to a higher center specializing in VL research (RMRIMS) for parasitological confirmation.

A comprehensive community-based information, education, and communication (IEC) strategy was implemented in areas of MSF-supported services from the start of the programme, with community and health providers education regarding the signs and symptoms of PKDL being an integral component.

For patients presenting with a history consistent with VL (splenomegaly and fever of >2 weeks duration) the diagnosis was confirmed with a rK39 rapid diagnostic test (DiaMed-IT LEISH; DiaMed AG, Cressier, Switzerland). Patients with a positive rK39 test result but a history of relapse, or those suspected to have VL despite a negative diagnostic test result were referred for splenic or bone marrow aspiration to confirm the presence of parasites.

All patients received four doses of 5 mg/kg Ambisome, given over 4–10 days depending on disease severity. Initially, all patients were given 5 mg/kg on days 0, 1, 4, and 9. However, due to the increasing number of patients and the limited capacity of the hospital, the treatment duration was reduced to 4 consecutive days at the hospital level for all clinically stable patients. Patients given ambulatory treatment at the primary health centers and clinically severe patients admitted to the hospital continued to receive the 7–10 day regimen. Patients were discharged as ‘initial cure’ once they completed treatment and exhibited resolution of fever, reduction of splenomegaly, and improvement of symptoms. A previous study using the same treatment regimen at the same site showed >98% effectiveness at 6 months [7], therefore spleen or bone marrow test-of-cure biopsy was planned only when treatment failure was suspected. However, no patients during the 5-year study period were suspected of treatment failure.

Basic demographic details, clinical history, and anthropometric measurements were recorded for all patients. Patients considered at higher risk of being HIV-positive (specifically migrants and those presenting with atypical presentations or relapses) were offered HIV testing, as were all patients who re-attended with confirmed PKDL. During treatment, dedicated trained health counselors gave all the patients health education about VL, including a detailed explanation, with pictures, of the possible manifestations of PKDL. Patients were counseled to return for review at 3, 6, and 12 months following treatment or earlier/later if any of these symptoms occurred.

Patients presenting with suspected PKDL were examined and the time of disease onset, distribution, and morphology of lesions recorded. All patients were referred for slit-skin smear examination (SSE). The largest and/or most prominent lesions were incised at the level of the dermis, samples taken and Giemsa stained for microscopic examination for *L. donovani* bodies.

**Figure 1:**
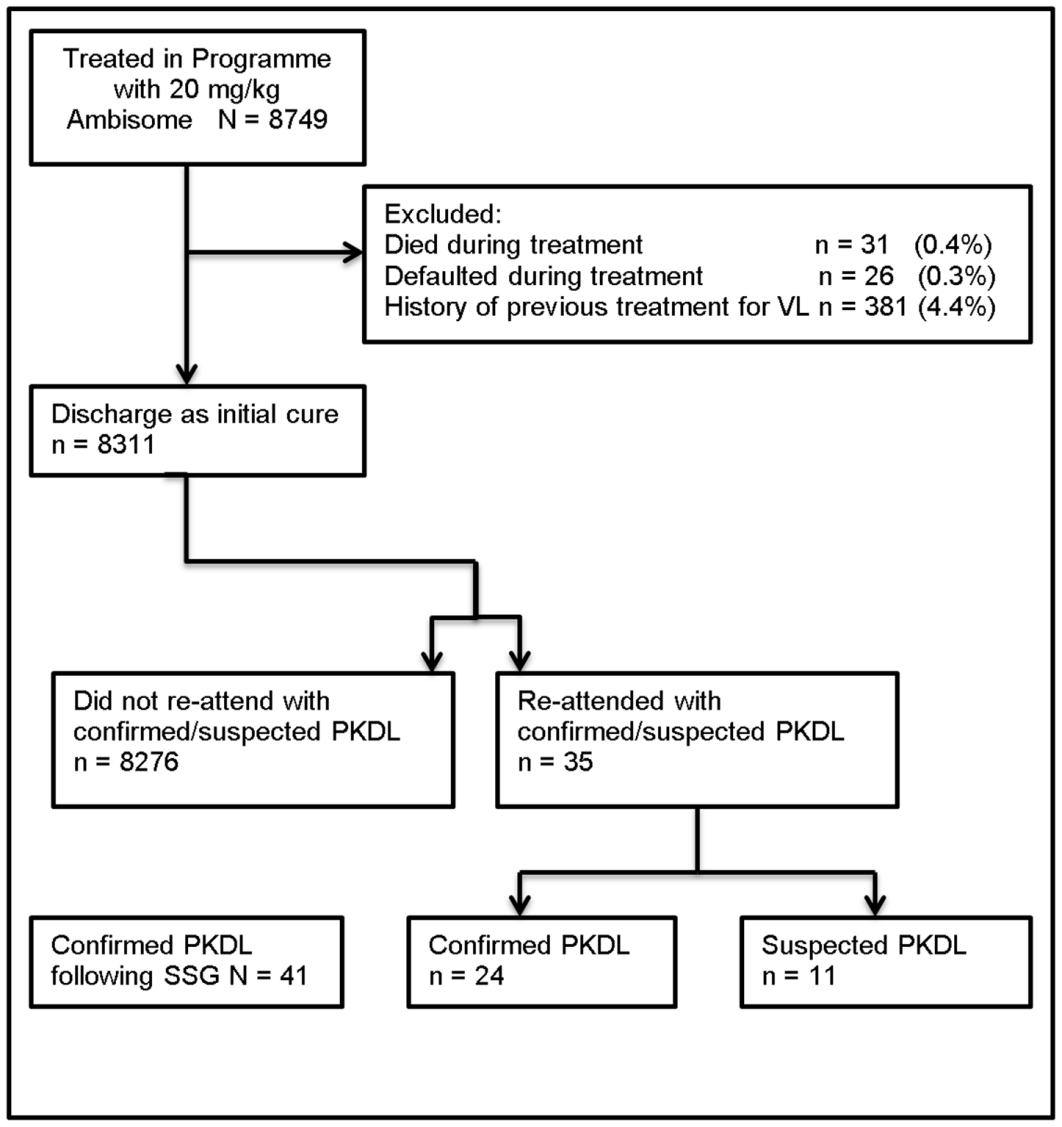
Flowchart of patients treated in the MSF programme who went on to develop post Kala-azar dermal leishmaniasis (PKDL) after treatment with Ambisome. SSG, sodium stibogluconate treatment; VL, visceral leishmaniasis.

For the purposes of this study, the characteristics of patients with confirmed parasites on SSE (confirmed PKDL) were compared with those of the remaining treated cohort to investigate any risk factors for developing PKDL. Patients with negative SSE were considered as ‘suspected PKDL’ and their characteristics recorded but excluded from the main comparative analysis with the remaining cohort. Patients with a history of relapse with or without previous treatment for VL were also excluded from the analysis (Figure 1).

Over the 5-year period of analysis (2007–2012), an additional 41 patients presented to the MSF programme with confirmed PKDL and gave a history of treatment with sodium stibogluconate (SSG) for an original episode of VL. These patients were mostly those who had been identified through IEC activities and referrals from other medical practitioners; the patients had received prior VL treatment with SSG in government or private facilities. Detailed information on their condition at the time of their original treatment was unavailable. A history of treatment with SSG was determined by patient recall of treatment lasting >2 weeks consisting of regular intramuscular injections following diagnosis with VL. As a secondary analysis, the profiles of the post-Ambisome confirmed PKDL cases were compared with this cohort of 41 PKDL cases with a previous treatment history of SSG.

Throughout the project, all data were entered into a Microsoft Excel database; double data-entry was not done. Regular database cleaning comprised checks for inconsistencies and source document verification was available where necessary. An epidemiologist ensured the database was well maintained and undertook regular quality audits on data transfer. World Health Organization (WHO) Anthro and Anthro-Plus software (Geneva, Switzerland) were used to calculate a weight-for-height Z-score for children aged <5 years and a BMI-for-age Z-score for those aged ≥5–19 years. A retrospective analysis of all routinely collected programme data was then conducted using SPSS version 19 (IBM, Chicago, USA).

### Ethics statement

This analysis met the Médecins Sans Frontières Institutional Ethics Review Committee's criteria for a study involving the analysis of routinely collected programme data. Although a new treatment in the Indian setting, the programme utilised a recognised treatment for VL and was run in coordination with the State Health Society through a memorandum of understanding, which is the usual procedure for NGOs operating in this context. All electronic data were analysed anonymously.

## Results

A total of 8749 patients were treated for VL with 20 mg/kg Ambisome between July 2007 and August 2012, with an in-programme mortality rate of 0.4% (n = 31) and a default rate of 0.3% (n = 26) (Figure 1). Of the 8692 patients completing treatment (discharged as initial cure), an additional 381 patients were excluded from the analysis due to presenting as a relapse having been treated with a drug other than Ambisome in the past. From the remaining 8311, a total of 24 (0.3%) patients re-attended the programme with symptoms of PKDL which were confirmed on SSE. All 24 patients were tested for HIV, of which one tested positive. A further 11 patients (0.1%) re-attended with symptoms and a history suspicious of PKDL, but their SSE results were negative.

There were no significant associations between patient demographics (sex, age group, caste, season of treatment), nor clinical characteristics (nutritional status, duration of illness prior to treatment, admission spleen size, change in spleen size by time of discharge, Hb, duration of treatment or HIV status) at time of initial treatment and the risk of developing confirmed PKDL. Neither did the characteristics of patients with confirmed or suspected PKDL and those of the cured cohort significantly differ.

**Figure 2:**
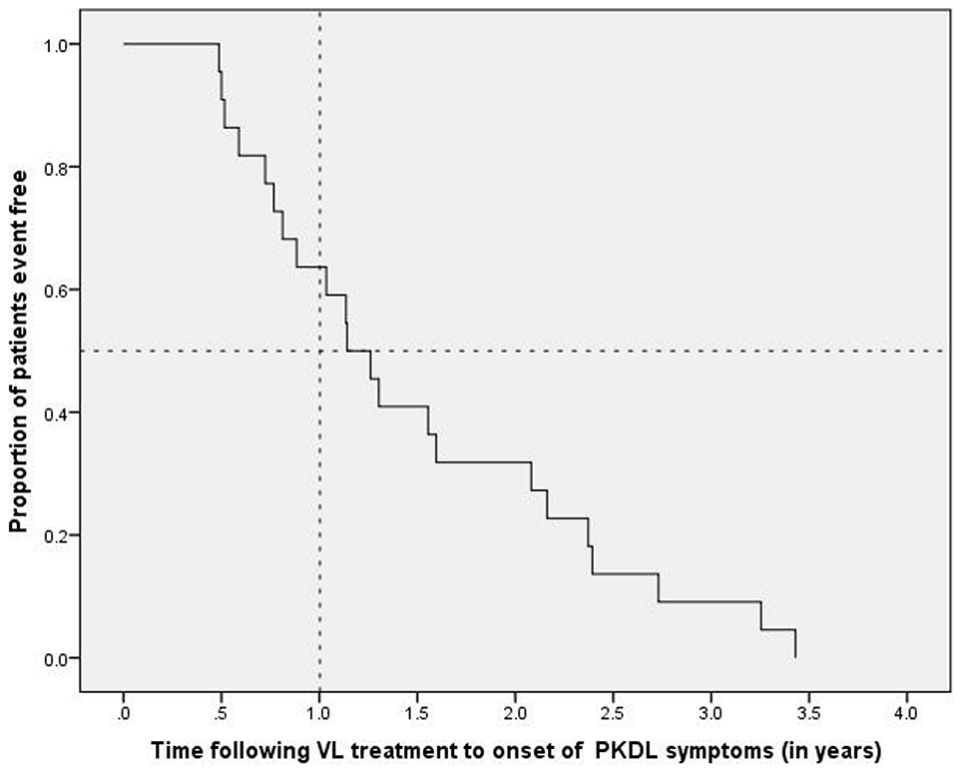
Uncensored Kaplan–Meier graph of time to developing post Kala-azar dermal leishmaniasis (PKDL) lesions following treatment of visceral leishmaniasis (VL) with 20 mg/kg Ambisome.

The mean ± standard deviation (range) time to the onset of lesions of confirmed PKDL as reported by the patients was 1.5±0.9 (0.5–3.4) years, with a median (interquartile range [IQR]) of 1.2 (0.8–2.2) years. However, the mean (range) time to confirmation of diagnosis of PKDL was longer at 2.4 (1.0–4.4) years, with a median (IQR) of 2.2 (1.7–3.0) years. The time to onset of PKDL lesions is shown in the Kaplan–Meier graph (Figure 2), where a Mantel–Cox log rank test of equality of survival distributions showed no significant difference between the sexes (p = 0.652). Of note, 68.2% of patients developed PKDL lesions within 2 years, with 36.4% of patients developing lesions <12 months following treatment (Table 1). Of the patients presenting with confirmed PKDL, females were significantly younger than males (mean age 14 vs 33 years, respectively; p = 0.048).

**Table 1:** Characteristics of patients with confirmed post-Ambisome PKDL stratified by sex (N = 24).

Characteristic		Male (%) n = 11	Female (%) n = 13	P Mann–Whitney U
Time to onset of lesions, years	<1	5 (45.5)	3 (23.1)	
	1–<2	1 (9.1)	6 (46.2)	
	2–<3	3 (27.3)	2 (15.4)	
	≥3	0	2 (15.4)	
	Missing	2 (18.2)	0	
	Median (IQR)	0.9 (0.7–2.3)	1.3 (0.8–2.2)	0.76
Time to formal diagnosis, years	<1	1 (9.1)	0	
	1 to <2	4 (36.4)	4 (30.8)	
	2 to <3	4 (36.4)	6 (46.2)	
	≥3	2 (18.2)	3 (21.3)	
	Median (IQR)	2.1 (1.5–2.9)	2.3 (1.7–3.0)	0.44
Age groups, years	<5	0	2 (15.4)	
	5 to <15	3 (27.3)	5 (38.5)	
	15 to <30	1 (9.1)	3 (23.1)	
	30 to <45	4 (36.4)	2 (15.4)	
	≥45	3 (27.3)	1 (7.7)	
	Median (IQR)	33 (14–45)	14 (11.5–25)	0.048
Type of lesion	Macular	10 (90.9)	11 (84.6)	
	Maculo-papular	0	2 (15.4)	
	Nodular-papular	1 (9.1)	0	

All data are n (%) unless stated otherwise.

IQR, interquartile range; PKDL, post-Kala-azar dermal leishmaniasis.

### Comparison of post-Ambisome PKDL with post-SSG PKDL

Over the 5-year period of analysis, 41 patients presented to the MSF programme with confirmed PKDL and a history of treatment with SSG for their original episode of VL (Table 2). The main differences between the post-Ambisome and post-SSG cohorts were time to onset/diagnosis of symptoms and lesion morphology. In patients with a history of SSG treatment, maculo-papular lesions were much more prevalent than in those treated with Ambisome, and the median time from treatment to onset of symptoms was significantly higher (2.9 years for SSG vs 1.2 years for Ambisome; p = 0.002).

**Table 2:** Comparison of PKDL patients following treatment with SSG and Ambisome.

	Confirmed PKDL following SSG (%) n = 41	Confirmed PKDL following Ambisome (%) n = 24	P Mann-Whitney U
**Time to onset of lesions, years**			
<1	6 (14.6)	8 (33.3)	
1 to <2 years	8 (19.5)	7 (29.2)	
2 to <3 years	6 (14.6)	5 (20.8)	
3 to <5 years	7 (17.1)	2 (8.3)	
≥5	13 (31.7)	-	
Missing	1 (2.4)	2 (8.3)	
Median (IQR)	2.9 (1.5–5.5)	1.2 (0.8–2.2)	0.002
**Time to formal diagnosis, years**			
<1	0	1 (4.2)	
1 to <2	3 (7.3)	8 (33.3)	
2 to <3	11 (26.8)	10 (41.7)	
3 to <5	3 (7.3)	5 (20.8)	
≥5	24 (58.5)	-	
Median (IQR)	5.1 (2.5–13.3)	2.2 (1.7–3.0)	<0.001
**Age groups, years**			
<5	0	2 (8.3)	
5 to <15	12 (29.3)	8 (33.3)	
15 to <30	16 (39.0)	4 (16.7)	
30 to <45	8 (19.5)	6 (25.0)	
≥45	5 (12.2)	4 (16.7)	
Median (IQR)	22 (13.5–30.5)	19 (12–33)	0.147
**Type of lesion**			
Macular	12 (29.3)	21 (87.5)	
Maculo-papular	21 (51.2)	2 (8.3)	
Papular	1 (2.4)	0	
Maculo-nodular	2 (4.9)	0	
Nodular-papular	1 (2.4)	1 (4.2)	
Missing	4 (9.8)	0	

All data are n (%) unless stated otherwise.

IQR, interquartile range; PKDL, post-Kala-azar dermal leishmaniasis; SSG, sodium stibogluconate treatment.

## Discussion

PKDL remains a challenging condition from all perspectives. The causes of PKDL are likely to be multifactorial [10], but there is no detailed understanding of its pathogenesis. Until recently it was assumed that PKDL occurred only after treatment for VL, but there is increasing evidence that PKDL can occur during VL or even before any clinical manifestation [9], [11]. The lack of simple and accurate diagnostic tools exacerbates the situation, particularly in resource-challenged areas. Currently in Bihar the only feasible method of confirming the diagnosis of PKDL is microscopic demonstration of *L. donovani* amastigotes from skin tissue aspirates. A study carried out in India found that 7–33% of macular PKDL lesions show amastigotes compared with 67–100% of nodular lesions [12]. This is of particular concern because a substantial proportion of cases of PKDL in the Indian subcontinent appear to be of macular morphology [9], [13], [14]. Moreover, the frequency of macular lesions may be underestimated as they are probably less likely to cause patients to seek treatment than the more disfiguring nodular and polymorphic lesions. Assays based on polymerase chain reaction have shown promising results and are more widely available in Nepal and Bangladesh [13], but this useful diagnostic tool is not routinely accessible in Bihar. Recent studies have suggested that serum immunoglobulin G levels could prove to be a useful tool in monitoring polymorphic PKDL incidence [15].

However, it is also important to reflect that the exclusion of parasite negative patients may bias this and other epidemiological analysis, which focus mainly on parasitologically confirmed PKDL. The recent WHO manual on PKDL management and control accepts a clinical case definition for initiation of treatment, and only recommends parasitological or molecular conformation in case of unresponsiveness to treatment [7]. Considering the lack of field diagnostic confirmatory tools and the high proportion of macular morphology patients, the practice of excluding parasite negative ‘suspected’ PKDL cases may inadvertently undermine the validity of current research into PKDL.

Several studies have described the characteristics of PKDL in the Indian subcontinent, but most are limited by small sample sizes and all describe patients mainly treated with SSG. Few studies have been able to identify any risk factors for developing PKDL. One retrospective cohort study in Nepal attempted to trace all patients who had received VL treatment in five endemic areas over a 10-year period to screen for PKDL. The study identified 2.4% of patients (16 of 680) with probable or confirmed PKDL and suggested that unsupervised treatment and inadequate SSG treatment in the past were significantly associated with PKDL; however clinical markers were not examined [14]. In the present study, all patients completed treatment with Ambisome and after examining clinical and demographic features no significant risk factors were identified.

The cohort of patients with VL described in the present study is the largest worldwide to date to have been treated with Ambisome, and comprises an estimated 5.8% of all reported VL cases in India between 2008 and 2011 [16]. Although clearly limited by passive re-attendance of patients with PKDL to the MSF programme, the regular arrival of patients with no history of contact with the programme suggests that the active community IEC strategy had some success in raising awareness and identifying PKDL cases in the wider community.

A particular strength of this study is the robust database that has been maintained throughout the programme and has minimal missing data. However, there are several limitations, which mean caution must be taking in interpreting the data. Despite comprehensive patient education during treatment, reliance on passive re-attendance by patients means that the recorded incidence of PKDL following treatment is very likely to be an underestimate. Additionally, the variability in time from VL treatment to presentation with PKDL makes the interpretation of incidence more difficult. For example, most SSG-treated patients present with PKDL >3 years following treatment, and as this period post-treatment has yet to elapse for most of the cohort treated with Ambisome, there remains the possibility of a biphasic incidence curve which could show a further peak after an increased period of follow-up. Also, the lack of sensitive diagnostic tools meant that the primary analysis was only determined for those patients with confirmed PKDL, excluding probable cases of PKDL. Finally, for the patients with PKDL who presented following treatment with SSG, it was not possible to confirm the accuracy of patient-reported timing of treatment and onset of symptoms.

It is worth emphasizing that the present analysis reflects a minimum estimate of PKDL incidence following treatment with 20 mg/kg Ambisome in Bihar. The incidence (0.3%) of confirmed PKDL cases found in the present study of primary VL patients is lower than that reported following treatment with mostly SSG in India [1] and Nepal [14], and much lower than what has been reported in Bangladesh [11]. The difference in incidence of PKDL within the subcontinent countries, despite the same pathogen and treatment histories, is of interest and worthy of further research. Indeed, the experience of MSF in Bangladesh has suggested that an estimated 10% of patients with VL proceed to develop PKDL after treatment with intravenous Ambisome 15 mg/kg, although this estimate includes probable cases and was based on active case detection (K. Ritmeijer, personal communication).

Most (>85%) patients with confirmed or suspected PKDL in the present study showed exclusively macular morphology, whereas in previous reports from India the proportion of patients with macular PKDL lesions following treatment with more established drugs has been estimated as 23% [17] and 48% [9]. This could be as a consequence of treatment with Ambisome, however the pattern is similar to that described in Bangladesh and Nepal [13], [14]. This might be explained by increased identification of ‘milder’ cases of PKDL through IEC at the community level - cases that may not have otherwise presented to health providers. Alternatively, it is possible that the development of PKDL following treatment with Ambisome is more likely to be of macular morphology, which could in turn be associated with a lower risk of transmission. If true, Ambisome could be considered a better therapeutic option considering the ultimate aim of disease elimination.

A particular area of difference between treatment with Ambisome and treatment with SSG was the median post-treatment time-to-onset of PKDL. At 1.2 years with Ambisome, this period appears much shorter than seen both in our comparison analysis with SSG-treated patients and than that reported from other studies. SSG and conventional amphotericin B are strong immunomodulators [18], [19], whilst Ambisome is thought to have additional capacity in down-regulating disease promoting suppressive cytokines [20]. Considering these results, immunoprofiling of patients following treatment with Ambisome may be helpful in identifying other risk factors of potential importance in the development of PKDL. Meanwhile, the WHO recommendation of a 10 mg single-dose liposomal amphotericin B dose and lower-dose combination therapies for the treatment of patients with VL in the Indian subcontinent [21] increases the importance of educating patients and monitoring outcomes with regards to PKDL.

In conclusion, this study demonstrates that in the Indian context, the development of PKDL following treatment for VL with 20 mg/kg liposomal amphotericin B (Ambisome) is a possibility. We suggest that it occurs after shorter time periods than might have been expected, although no risk factors for its development were identified.

## Supporting Information

Table S1 Demographic characteristics of patients with confirmed or suspected PKDL at initial treatment compared with the remaining cohort.
(DOCX)

Table S2 Clinical characteristics of patients with confirmed or suspected PKDL at initial treatment compared with remaining cohort.
(DOCX)

Checklist S1 STROBE Checklist.
(DOC)
