# Supplementary material for: Post Kala-Azar Dermal Leishmaniasis following Treatment with 20 mg/kg Liposomal Amphotericin B (Ambisome) for Primary Visceral Leishmaniasis in Bihar, India
Source: PLoS Negl Trop Dis. 2014 Jan 2;8(1):e2611. doi: 10.1371/journal.pntd.0002611 (PMC3879248; doi:10.1371/journal.pntd.0002611)
Supplement: Table S1 — Demographic characteristics of patients with confirmed or suspected PKDL at initial treatment compared with the remaining cohort. (DOCX) [file pntd.0002611.s001.docx]

| **Characteristic** | **Risk factor** | **Did not reattend with PKDL** | **Confirmed PKDL patients** | **Suspected PKDL patients** | **Unadjusted OR: confirmed PKDL vs did not reattend with PKDL (95% CI)** | **p** |
| --- | --- | --- | --- | --- | --- | --- |
| Sex (n = 8311) |  | **n = 8276** | **n = 24** | **n = 11** |  |  |
|  | Female | 3610 (43.6) | 13 (54.2) | 7 (63.6) | 1.5 (0.7–3.4) | 0.30 |
|  | Male | 4666 (56.4) | 11 (45.8) | 4 (36.4) | – |  |
| Caste^a,b^ (n = 8267) |  | **n = 8232** | **n = 24** | **n = 11** |  |  |
|  | Scheduled caste/tribe | 2418 (29.4) | 5 (20.8) | 3 (27.3) | 0.5 (0.1–1.8) | 0.29 |
|  | Other backward class | 4567 (55.5) | 14 (58.3) | 6 (54.5) | 0.8 (0.3–2.1) | 0.61 |
|  | General category | 1247 (15.1) | 5 (20.8) | 2 (18.2) | – |  |
| Age groups, years  (n = 8311) |  | **n = 8276** | **n = 24** | **n = 11** |  |  |
|  | <5 | 573 (6.9) | 3 (12.5) | 0 | 2.5 (0.4–14.7) | 0.21 |
|  | 5 to <15 | 3145 (38.0) | 9 (37.5) | 6 (54.5) | 1.4 (0.4–6.0) | 0.78 |
|  | 15 to <30 | 1899 (22.9) | 4 (16.7) | 3 (27.3) | – |  |
|  | 30 to <45 | 1496 (18.1) | 4 (16.7) | 2 (18.2) | 1.3 (0.2–6.8) | 0.74 |
|  | ≥45 | 1163 (14.1) | 4 (16.7) | 0 | 1.6 (0.3–8.8) | 0.49 |

Table 3. Demographic characteristics of patients with confirmed or suspected PKDL at initial treatment compared with the remaining cohort

| Season of treatment (n = 8311) |  | **n = 8276** | **n = 24** | **n = 11** |  |  |
| --- | --- | --- | --- | --- | --- | --- |
|  | March–June | 3535 (42.7) | 8 (33.3) | 2 (18.2) | 0.6 (0.2–2.0) | 0.37 |
|  | July–October | 2468 (29.8) | 8 (33.3) | 4 (36.4) | 0.9 (0.3–2.5) | 0.87 |
|  | November–February | 2273 (27.5) | 8 (33.3) | 5 (45.5) | – |  |

All data are n (%) unless stated otherwise.

^a^Data were missing or incorrectly coded on database.

^b^‘Scheduled caste/tribe’ are terms used for two groups of historically disadvantaged people recognized in the Constitution of India. ‘Other backward class’ is a collective term used by the government of India for castes that are educationally and socially disadvantaged. Combined they account for approximately 60% of India’s population. The ‘General category’ comprises the remainder of the population.

OR, odds ratio; PKDL, post Kala-azar dermal leishmaniasis.
