# Supplementary material for: Post Kala-Azar Dermal Leishmaniasis following Treatment with 20 mg/kg Liposomal Amphotericin B (Ambisome) for Primary Visceral Leishmaniasis in Bihar, India
Source: PLoS Negl Trop Dis. 2014 Jan 2;8(1):e2611. doi: 10.1371/journal.pntd.0002611 (PMC3879248; doi:10.1371/journal.pntd.0002611)
Supplement: Table S2 — Clinical characteristics of patients with confirmed or suspected PKDL at initial treatment compared with remaining cohort. (DOCX) [file pntd.0002611.s002.docx]

Table 4. Clinical characteristics of patients with confirmed or suspected PKDL at initial treatment compared with remaining cohort

| **Characteristic^a^** | **Risk factor** | **Did not reattend with PKDL (%)** | **Confirmed PKDL patients (%)** | **Suspected PKDL patients (%)** | **Unadjusted OR: confirmed PKDL vs did not reattend with PKDL (95% CI)** | **p** |
| --- | --- | --- | --- | --- | --- | --- |
| Nutritional status (n = 6834) |  | **n = 6804** | **n = 21** | **n = 9** |  |  |
|  | Severe acute malnutrition | 1235 (18.2) | 1 (4.8) | 2 (22.2) | 0.2 (0.01–1.5) | 0.22 |
|  | Moderate acute malnutrition | 1563 (23.0) | 6 (28.6) | 2 (22.2) | 1.1 (0.4–2.9) | 0.85 |
|  | Normal | 4006 (58.9) | 14 (66.7) | 5 (55.6) | – |  |
| Time ill prior to treatment, weeks (n = 8310) |  | **n = 8275** | **n = 24** | **n = 11** |  |  |
|  | ≤4 | 4796 (58.0) | 14 (58.3) | 6 (54.5) | – |  |
|  | >4 to ≤8 | 2057 (24.9) | 7 (29.2) | 3 (27.3) | 1.2 (0.5–2.9) | 0.74 |
|  | >8 | 1422 (17.2) | 3 (12.5) | 2 (18.2) | 0.7 (0.1–2.6) | 0.78 |

| Spleen size at admission, cm (n = 8307) |  | **n = 8272** | **n = 24** | **n = 11** |  |  |
| --- | --- | --- | --- | --- | --- | --- |
|  | <3 | 1233 (14.9) | 3 (12.5) | 1 (9.1) | – |  |
|  | 3–6 | 4270 (51.6) | 13 (54.2) | 9 (81.8) | 1.3 (0.3–6.9) | 1.0 |
|  | >6 | 2769 (33.5) | 8 (33.3) | 1 (9.1) | 1.2 (0.3–7.0) | 1.0 |
| Change in spleen size by discharge, cm/day (n = 8281) |  | **n = 8246** | **n = 24** | **n = 11** |  |  |
|  | <0.5 | 4633 (56.2) | 12 (50) | 5 (45.5) | 0.8 (0.3–1.7) | 0.54 |
|  | ≥0.5 | 3613 (43.8) | 12 (50) | 6 (54.5) | – |  |
| Hemoglobin level, g/dL  (n = 8288) |  | **n = 8253** | **n = 24** | **n = 11** |  |  |
|  | <6 | 1055 (12.8) | 3 (12.5) | 1 (9.1) | 0.8 (0.1–3.9) | 1.0 |
|  | 6–8 | 2735 (33.1) | 7 (29.2) | 6 (54.5) | 0.8 (0.3–2.2) | 0.61 |
|  | 8–10 | 2694 (32.6) | 8 (33.3) | 4 (36.4) | 0.9 (0.3–2.5) | 0.81 |
|  | >10 | 1769 (21.4) | 6 (25.0) | 0 (0) | – |  |
| Length of treatment, days (n = 8294) |  | **n = 8259** | **n = 24** | **n = 11** |  |  |
|  | 4 | 1567 (19.0) | 4 (16.7) | 1 (9.1) | 0.9 (0.2–2.6) | 1.0 |
|  | 7–10 | 6692 (81.0) | 20 (83.3) | 10 (90.9) | – |  |
| HIV status (n = 8311) |  | **n = 8276** | **n = 24** | **n = 11** |  |  |
|  | Positive | 95 (1.1) | 1 (4.2) | 0 | 3.7 (0.1–23.5) | 0.24 |
|  | Negative | 8181 (98.9) | 23 (95.8) | 11 (100) | – |  |

All data are n (%) unless stated otherwise.

^a^Where patient numbers are <8311, remaining data were missing or incorrectly coded on database.

OR, odds ratio; PKDL, post Kala-azar dermal leishmaniasis.
